# A sparse coding model of V1 produces surround suppression effects in response to natural scenes

**DOI:** 10.1186/1471-2202-14-S1-P335

**Published:** 2013-07-08

**Authors:** Allison Del Giorno, Mengchen Zhu, Christopher J Rozell

**Affiliations:** 1 Electrical and Computer Engineering, Georgia Institute of Technology, Atlanta, GA 30332, USA; 2 Biomedical Engineering, Georgia Institute of Technology, Atlanta, GA 30332, USA

## 

While many neural coding models have been proposed for V1, it remains an open question as to which model best describes the diversity of observed response properties. For instance, the canonical linear-nonlinear model (LN) partially explains some fundamental mechanistic and phenomenological properties of V1, but is unable to explain many nonlinear response properties that are likely associated with the keys to efficient and robust human vision.

**Figure 1:**
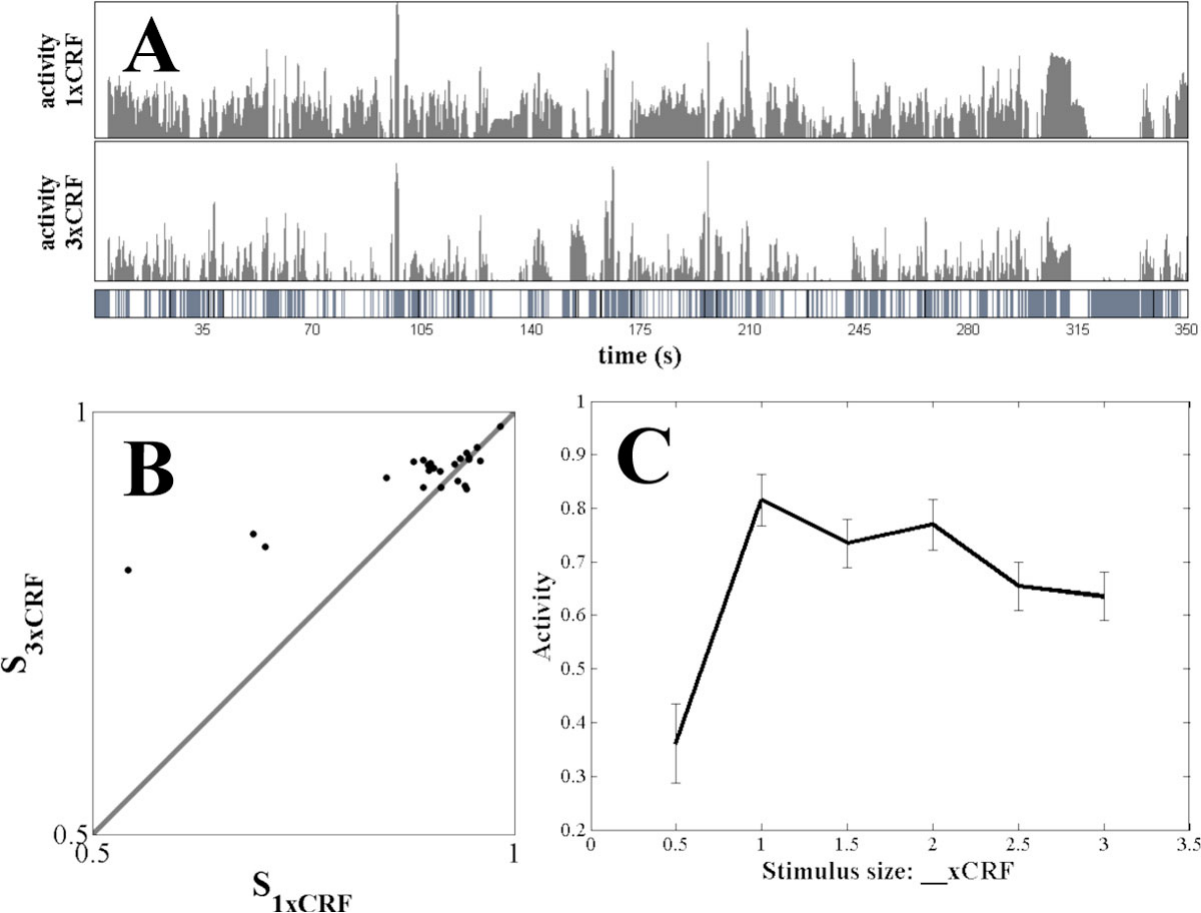
**Surround suppression effects in a sparse coding model (compare to **[1,2]**and results with white noise stimuli, not shown)**. **A**. A single cell's responses to natural movies masked to 1x (top plot) and 3x (middle plot) the CRF size. The bottom bar marks frames with significant suppression (white), facilitation (black), or no significant change (gray) when the nCRF is included. **B**. Lifetime sparseness of each cell with 1xCRF stimulus (x-axis) and 2xCRF stimulus (y-axis). Sparsity increases with inclusion of the nCRF. **C**. Population average of the mean activity of each cell with increasing stimulus size.

For example, surround suppression is one such nonlinear response property in which visual stimuli extending beyond the classical receptive field (CRF) selectively diminish neural responses. This property has been studied through electrophysiology experiments with synthetic stimuli (e.g., gratings). Surprisingly, high level sparse coding models implemented in a biologically plausible dynamical system have been shown to produce surround suppression effects that match individual and population observed responses [3]. More recently, surround suppression has been investigated experimentally using natural stimuli, and these experiments have shown an increase in the sparsity of measured responses [1,2]. Despite these findings, it remains unclear whether a functional sparse coding model is sufficient to produce the types of surround suppression observed with natural stimuli. In this abstract, we demonstrate that the surround suppression effects recently observed with natural stimuli are also emergent properties of a sparse coding model (Figure 1).
